# *In vitro* synthesis of a Major Facilitator Transporter for specific active transport across Droplet Interface Bilayers

**DOI:** 10.1038/srep39349

**Published:** 2016-12-20

**Authors:** Heather E. Findlay, Nicola J. Harris, Paula J. Booth

**Affiliations:** 1 Department of Chemistry, Kings College London, Britannia House, 7 Trinity Street, London, SE1 1DB, UK

## Abstract

Nature encapsulates reactions within membrane-bound compartments, affording sequential and spatial control over biochemical reactions. Droplet Interface Bilayers are evolving into a valuable platform to mimic this key biological feature in artificial systems. A major issue is manipulating flow across synthetic bilayers. Droplet Interface Bilayers must be functionalised, with seminal work using membrane-inserting toxins, ion channels and pumps illustrating the potential. Specific transport of biomolecules, and notably transport against a concentration gradient, across these bilayers has yet to be demonstrated. Here, we successfully incorporate the archetypal Major Facilitator Superfamily transporter, lactose permease, into Droplet Interface Bilayers and demonstrate both passive and active, uphill transport. This paves the way for controllable transport of sugars, metabolites and other essential biomolecular substrates of this ubiquitous transporter superfamily in DIB networks. Furthermore, cell-free synthesis of lactose permease during DIB formation also results in active transport across the interface bilayer. This adds a specific disaccharide transporter to the small list of integral membrane proteins that can be synthesised via *in vitro* transcription/translation for applications of DIB-based artificial cell systems. The introduction of a means to promote specific transport of molecules across Droplet Interface Bilayers against a concentration gradient gives a new facet to droplet networks.

Biological membranes are complex environments, with both integral and peripheral membrane proteins surrounded by bilayers composed of many different types of lipid. To study membrane protein function *in vitro* or to utilise them in synthetic biological systems it is generally necessary to extract the protein from this native membrane and reconstitute it into a detergent or lipid system, the properties of which can be more readily controlled. A variety of lipid structures have been used to replicate the membrane environment; from bicelles^1^,^2^ and nanodiscs^3^,^4^ to larger liposomes and giant unilamellar vesicles^5^,^6^. These vesicles provide a self-contained inner compartment that allows for the study of movement of molecules across the bilayer and are increasingly used as drug delivery systems in the medical field^7^. A more recent development is the Droplet Interface Bilayer (DIB). In this system sub-microlitre aqueous droplets are submerged in an oil phase, with each droplet surrounded by a lipid monolayer. When two droplets are brought together, they spontaneously zip up to form a stable bilayer area at the point of contact^8^,^9^. DIBs have several advantages for the study of membrane proteins; smaller amounts of protein are required than for traditional planar lipid bilayer techniques and DIBs are particularly useful for the analysis of bilayer asymmetry. Although the first examples of DIBs involved only two droplets, manually manipulated, microscale technologies are transforming these systems. Microfluidics devices and 3D printing techniques that allow precise control of the contents and flow rate during droplet formation have been used to make DIBs networks of defined size, composition and spatial distribution that could provide a promising platform for further synthetic biology applications and the development of synthetic tissues^10^,^11^,^12^.

The clear spatial compartmentalisation between droplets provides a prospective method of controlling chemical reactions and biological processes in synthetic *in vitro* systems. In order to realise the potential of DIBs in synthetic devices the bilayers have to be functionalised. Self-inserting toxins, together with integral membrane channel and transport proteins provide a means to regulate transport and information exchange across the DIB. To date the main protein harnessed in DIBs has been the toxin, α-haemolysin which spontaneously inserts into membranes to give a large, non-selective pore^13^,^14^. There have been very few uses of integral membrane proteins in DIBs. Existing examples have predominantly been channels, including the mechanosensitive channel MscL that provides a large non-selective pore^15^. Ion channels such as K^+^ channels have been used enabling flow of specific ions across the DIB^16^ and the light-driven proton pump bacteriorhodopsin has been successfully used to produce a proton gradient across DIBs, in response to illumination^17^. In addition to controlling ion flow, however, to exploit DIB systems for compartmentalised chemistry or artificial cells it is desirable to have a more specific method of moving a large variety of small molecules between droplets in a controlled manner. Harnessing natural membrane transport proteins would provide an ideal solution. It remains to be demonstrated that small molecule and metabolite transporters can be efficiently integrated in DIBs to regulate specific transport across the bilayer. Moreover, in order to utilise droplets separated by DIBs as nanoreactors, the transport of reactants/products across the DIB must be sufficient to enable efficient reactions within the droplets.

The Major Facilitator Superfamily (MFS) is a large and diverse family of secondary transporters with over 10,000 sequenced members including those that show specificity for sugars, amino acids, neurotransmitters and drugs^18^, making them good targets for transport in DIBs. The 3-dimensional crystal structures of several of these proteins have been determined, such as the clinically important human glucose transporter GLUT1^19^, which displays a common fold composed of 12 transmembrane α helices divided into two 6-helix bundles with the substrate binding site in the middle of the bilayer at the domain interface. One of the best characterised MFS proteins is lactose permease (LacY), the H^+^/lactose transporter from *E. coli*.^20^. Its substrate specificity is well understood, binding and transporting a range of small sugar-based molecules that contain a galactosyl rather than a glucosyl ring^21^. It has been purified and reconstituted into liposomes and giant unilamellar vesicles^5^,^22^ and its mechanism has additionally been studied by molecular dynamics simulations as well as experiments^23^. Transport activity is known to be strongly influenced by the surrounding bilayer environment^24^,^25^,^26^, raising the possibility of tuning the protein’s properties depending on the lipids chosen to form the droplet monolayers. In this study we use LacY as a model protein for the incorporation of a secondary transporter into a Droplet Interface Bilayer system, using both exogenously expressed, reconstituted protein and *in vitro* transcription/translation to synthesise protein in the droplet.

## Results

### Active secondary transport in a DIB system

**Figure 1:**
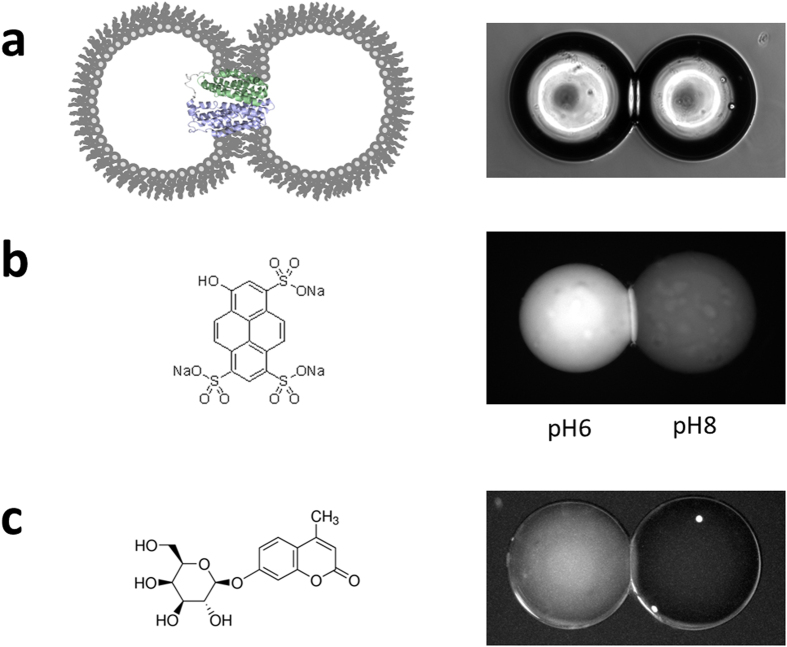
Assembling a Droplet Interface Bilayer system for the transport of small sugars through LacY. (**a**) Schematic of a DIB with LacY incorporated (left) with a brightfield image of two droplets forming the bilayer at the point of contact (right panel). (**b**) The pH sensitive dye pyranine (left) was loaded in two droplets at a concentration of 50 μM dye in 50 mM sodium phosphate buffer of two different pH. The image shown on the right was taken after 1 hour using a fluorescent microscope. (**c**) The fluorescent sugar MUG (left) is a substrate of LacY. A DIB was formed from DOPC lipids (right panel). The left hand side droplet was loaded with 50 μM MUG while the right hand side droplet contained only buffer. The image shown was taken after 1 hour.

The basic components of a system for sugar transport in DIBs are shown in Fig. 1. Two individually formed aqueous droplets, in a hexadecane solvent background, are each surrounded by a monolayer of lipids, with the transporter protein inserted into the bilayer region between the compartments. The droplets and the bilayer region that forms are easily visualised by microscopy (Fig. 1a). In most MFS transporters active transport is driven by an electrochemical gradient, a proton gradient in the case of LacY. Therefore it is important that the bilayer can maintain a pH difference over time. To test this, droplets were made composed of the lipid 1,2-dioleoyl-*sn*-glycero-3-phosphocholine (DOPC), a commonly used lipid that forms fluid bilayers at room temperature and has been previously used to make DIBs^15^. Its physical properties are well-characterised and it has been used extensively in *in vitro* systems to study membrane protein folding and activity. The dye pyranine exhibits a pH-dependent absorption shift, resulting in increased fluorescence intensity at a lower pH when excited with ultraviolet light. Two droplets were prepared with 50 μM pyranine, buffered with sodium phosphate to pH6 and pH8. When the droplets were imaged on a fluorescence microscope with a DAPI excitation and emission filter set (Fig. 1b), the pH difference could be observed by the increased fluorescence in the low pH droplet. After the DIB was formed, the difference in fluorescence between the two droplets was maintained over several hours, indicating no significant leakage of protons across the DIB, with any proton seepage being successfully buffered and the proton gradient maintained. DIB measurements were performed at 25 °C. In order to monitor transport across the bilayer, the fluorescent sugar 4-methylumbelliferyl-β-galactopyranoside (MUG) was used as a substrate of LacY^27^, allowing for the direct measurement of the movement of the molecule from one compartment to the other. To control for background leakage, two droplets were prepared from DOPC vesicles with MUG added to only one. After a DIB was formed (Fig. 1c) the fluorescence was confined entirely to the original droplet for >2 hour, demonstrating no detectable level of transport in the absence of protein.

**Figure 2:**
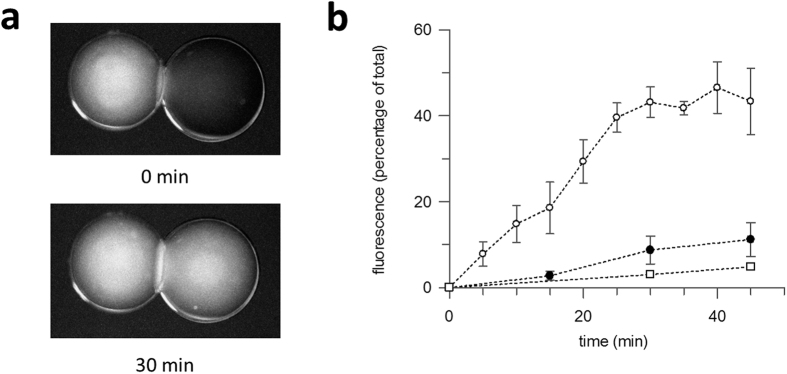
Active transport of the fluorescent sugar 4-methylumbelliferyl-b-galactopyranoside (MUG) by LacY incorporated into in a DOPC DIB. (**a**) Images taken at 0 mins and 30 mins are shown, that visualise transport of MUG across the bilayer. (**b**) MUG transported across the DIB was measured by integrating the signal from each droplet and expressing the fluorescence within the acceptor droplet (on the right hand side in a) as a percentage of total fluorescence. Transport of MUG with wild-type LacY plus a pH gradient (open circle), wild-type LacY without a pH gradient (closed circle), C154G mutant with a pH gradient (open square). Errors are ± SD of n ≥ 3.

There are two principal methods for adding purified membrane proteins to a DIB system, both starting from protein isolated in detergent solution. The “lipid-out” method involves submerging droplets of aqueous buffer into lipids dissolved in solvent. To incorporate the protein, the detergent is diluted below the critical micellar concentration in the aqueous droplets, in order to promote protein insertion without disrupting the lipid monolayer^8^. This proved an unreliable method for LacY, with either a lag time of up to an hour before any transport by LacY was observed, or a complete absence of transport, presumably due to protein aggregating in the droplet instead of incorporating in the bilayer. Therefore the alternative “lipid-in” method was used. Here the protein is pre-reconstituted into liposomes, then droplets are made directly from the liposome solution added to solvent, with the monolayer this time formed by fusion of the proteo-liposomes bilayers with the edge of the droplet. LacY was reconstituted into 100 nm vesicles composed of DOPC. DOPC proteo-liposomes were diluted into pH 7.5 buffer and a 400 nL droplet added to a hexadecane filled dish. A second droplet was made from empty DOPC vesicles diluted into pH 6.5 buffer containing MUG. The droplets were joined to create the bilayer and images taken at five minute intervals using a fluorescence microscope. An example of LacY transport in the resulting DIB is shown in Fig. 2a. The MUG started entirely within the left droplet of Fig. 2a and was transported to the right over time. The fluorescence was quantified by integrating the density and plotting the increasing signal in the right-hand droplet as a percentage of the total fluorescence (Fig. 2b, open circles). The fluorescence continued to increase until an equilibrium point of just below 50% after 30–40 mins. Although LacY requires a proton gradient for active transport, substrates can still be transported down a concentration gradient in its absence by facilitated diffusion^20^. DIBs were formed from proteo-liposomes with both droplets at pH7.5 (Fig. 2b, closed circles). Although transport was still detected, the amount of substrate transported was significantly less, with only ca. 10% of total fluorescence transported after 45 mins. As an additional control, the LacY mutant C154G was purified and reconstituted into liposomes. C145G-LacY is an inactive mutant that binds but does not transport substrates of LacY^28^. DIBs assembled using this mutant showed no substantial transport of MUG, even in the presence of a pH gradient (Fig. 2b, open squares).

### Manipulating the DIB lipid composition

**Figure 3:**
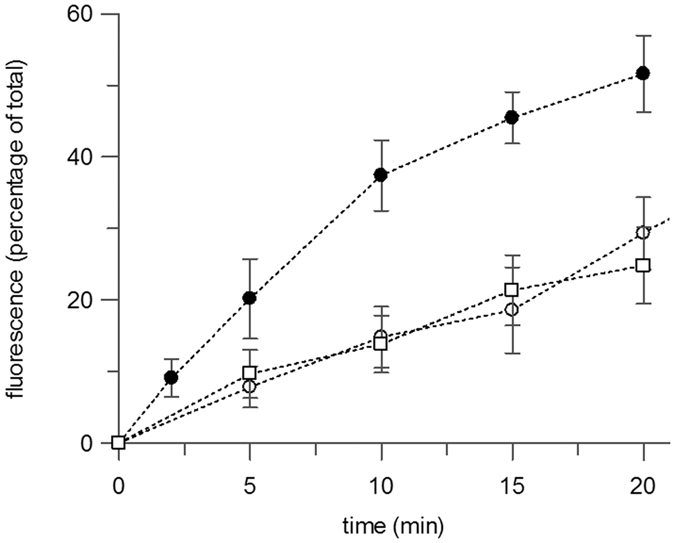
DIB lipid composition affects transport activity. MUG transported by LacY in DIBs composed of 100% DOPC (open circles), 80:20 DOPC:DOPG (open square) and 40:40:20 DOPC:DOPE:DOPG (closed circle), expressed as a percentage of the total fluorescence in the acceptor droplet. Errors are ± SD of n ≥ 3.

The composition of the lipid bilayer environment has a profound effect on membrane proteins in general^29^ and on the activity of LacY in particular^24^,^30^. Manipulating the lipids present in the DIBs is a potential route to modulating function, whilst maintaining the reproducibility and control of a fully synthetic system. Droplets were therefore made with different lipid mixes to establish the impact on both the DIBs themselves and protein activity. Although the DOPC used here is a neutral lipid, the native *E. coli* membrane of LacY contains phosphoglycerol headgroup lipids, as well as some cardiolipin, giving an overall negative charge to the membrane. Charge was introduced into the DIBs system by forming droplets with a mixture of DOPC and 1,2-dioleoyl-*sn-*glycero-3-phosph-(1’-*rac*-glycerol) (DOPG) lipids. High concentrations of DOPG were not suitable for this transport assay, as a 50 or higher mole percentage of DOPG resulted in bilayers that could not sustain the proton gradient for a sufficient length of time (data not shown). DIBs were made using a molar ratio of 80:20 DOPC:DOPG to give an overall charge close to that of the native membrane. This degree of charge resulted in stable DIBs that could hold a pH gradient for several hours. The transport of MUG by LacY was the same between these droplets as between those with only DOPC (Fig. 3).

The other key difference with the native membrane is that *E. coli* lipids contain a high percentage of non-lamellar lipids, predominantly with phosphoethanolamine headgroups. Substituting DOPC for 1,2-dioleoyl-*sn-*glycero-3-phosphoethanolamine (DOPE) lipids increases the curvature of the bilayer and the lateral pressure within the central hydrophobic region, while decreasing the lateral pressure in the headgroup region^31^. Making DIBs with native-like concentrations (60–70% DOPE) resulted in rapid fusing of the droplets and was therefore unsuitable. Instead LacY was reconstituted into vesicles composed of a molar ratio of 40:40:20 DOPC:DOPE:DOPG. DIBs assembled using this lipid mixture were stable for at least an hour, although typically with a smaller interface area than observed with DOPC only, or 80:20 DOPC:DOPG. In spite of this, LacY-mediated transport was significantly improved with DOPE present, reaching 50% of total fluorescence transported in 15–20 mins (Fig. 3, closed circle), approximately twice as fast as in droplets without DOPE. Assuming all the protein from the droplet was integrated into the interface, this would equate to a transport rate for MUG of 6–8 nmol/min/mg. In practice, only a proportion of LacY is likely to be incorporated, so this figure will underestimate the true rate of transport. This increase in transport rates as the bilayer environment more closely resembles the *E. coli* membrane is consistent with the known properties of LacY^32^.

**Figure 4:**
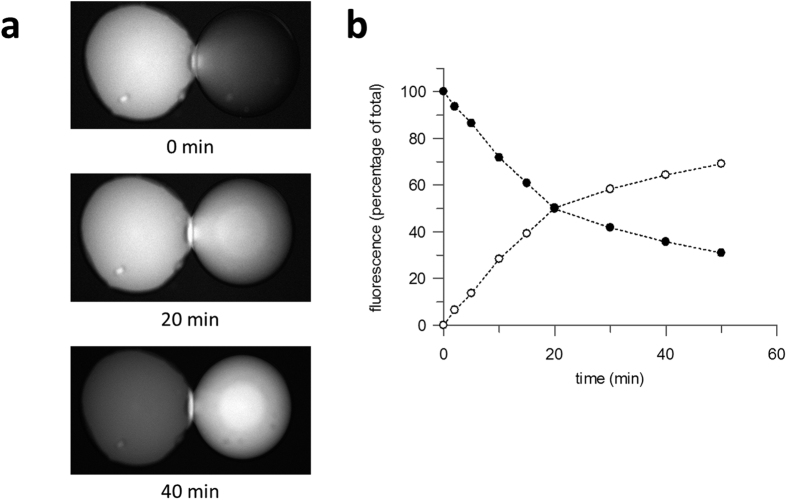
Uphill transport of MUG by LacY in a 40:40:20 (mole ratio) DOPC:DOPE:DOPG DIB. (**a**) Images taken of a DIB at 0 mins, 20 mins and 40 mins are shown, with fluorescence substrate, MUG, loaded into left hand droplet. (**b**) Relative fluorescence of donor (closed circle) and acceptor (open circle) droplets over time.

In the cell environment, the active transport stimulated by the proton gradient allows the substrate to be accumulated within the cell up its concentration gradient. Such active transport was also observed in the 40:40:20 DOPC:DOPE:DOPG DIBs (Fig. 4). The fluorescence increase in the right-hand droplet (Fig. 4b, open circles) and decrease in the left-hand droplet (closed circles) crossed over after 20 mins, then continued with a reduced rate of change. Thus LacY in DIBs is capable not only of accelerated transport, where the proton gradient catalyses substrate transport, but further able to drive true uphill transport where substrate is accumulated against its concentration gradient, as is the case *in vivo*.

### Cell-free expression in DIBS

Cell-free expression systems are being increasingly used for the production of proteins and to study their fold and function, including some membrane proteins. However, testing the activity of non-enzymatic proteins produced this way can be difficult, due to the difficulty and cost of scaling up from the relatively small amount of protein expressed. The very small volume used in DIB systems could allow for a new approach. In order to test the feasibility of such *in vitro* expression for secondary transporters in DIBs, droplets were prepared containing a commercially available (PURExpress) cell-free expression system that contains all the ribosomes, polymerases, NTPs, etc required for protein synthesis, the *lacy* plasmid DNA and liposomes, all buffered to pH 7.6. The PURExpress system has previously been used successfully in droplets, to express the Kcv potassium channel membrane protein, as well as water-soluble proteins and α-haemolysin^33^,^34^.

**Figure 5:**
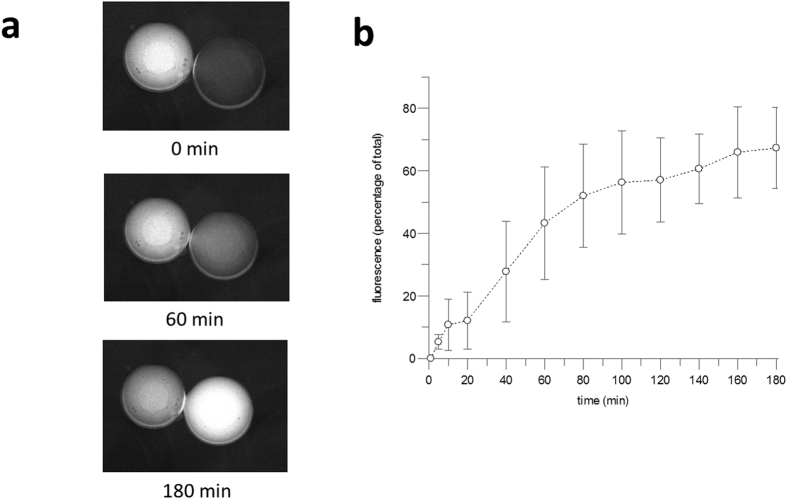
*In situ* cell-free expression and activity of LacY in 25:50:25 DOPC:DOPE:DOPG lipids. (**a**) Images taken of a DIB at 0 mins. 60 mins and 180 mins are shown. (**b**) MUG transported across the bilayer interface expressed as the percentage of fluorescence in the acceptor droplet. Errors are ± SD of n ≥ 3.

In order to assess cell-free synthesis of LacY, DIBs were formed with droplets containing liposomes and MUG buffered to pH 6.6 to form the pH gradient. Another MFS protein, GalP, has previously been shown to insert and fold poorly from an unfolded state into lipid bilayers containing high DOPC, therefore the lipid composition chosen for the cell-free expression was a molar ratio of 25:50:25 DOPC:DOPE:DOPG, intended to promote both transport activity and good insertion/folding of expressed protein. In order to form stable DIBs with these more complex aqueous solutions it was necessary to increase the lipid concentration from 1 to 5 mg/ml (w/v). DOPC:DOPE:DOPG DIBs are stable at room temperature but become unstable at higher temperatures. However, the PURExpress system only makes low amounts of protein at room temperature, with slower rates of expression than observed at 30 °C or above^35^. Thus, we followed the previous protocol of pre-incubating the cell-free reaction at 37 ^0^C in the presence of liposomes, prior to lowering the temperature to form first the droplet submerged in solvent, before assembling into a DIB^34^. *In vitro* cell-free expression systems produce protein at slower rates than *in vivo*, with the elongation rate of PURExpress at 37 °C being 0.5 amino acids per second^36^. For the earlier Kcv channel synthesis, PURExpress was pre-incubated at 37 °C for 1 hour, during which time the 94 amino acid subunit of Kcv will have been synthesised and inserted into the liposomes in the droplet, prior to forming a DIB. Here we pre-incubated PURExpress for 5 min at 37 °C to initiate transcription^37^ and then lowered the temperature for DIB formation which took a subsequent 5–10 mins at room temperature (5 mins for monolayer formation at room temperature and a maximum of 5 mins to bring the droplets together). This 5 min pre-incubation was sufficient to produce a significant increase in yield of LacY in a 2 hour room temperature cell-free reaction compared to a room temperature incubation alone (see SI Fig. 1). The 429 amino acid long LacY takes ~15 minutes to synthesise by PURExpress at 37 °C, and thus synthesis of full length LacY will not have occurred during the 10–15 min cell-free initiation/DIB formation time, especially since the temperature is lowered to 25 °C after 5 mins which slows translation. LacY most likely inserts during translation, which occurs before, during and after DIB formaton, with LacY inserting into the liposomes within the droplet, or possibly directly into the DIB itself. Figure 5 shows that MUG was transported across the interface, demonstrating the successful expression and insertion of LacY. The rate of transport was both slower and more variable than with reconstituted protein with at least an hour passing post DIB assembly before 50% of the substrate was transported. Crucially, uphill transport was still observed, confirming protein in the interface was fully functional.

## Discussion

**Figure 6:**
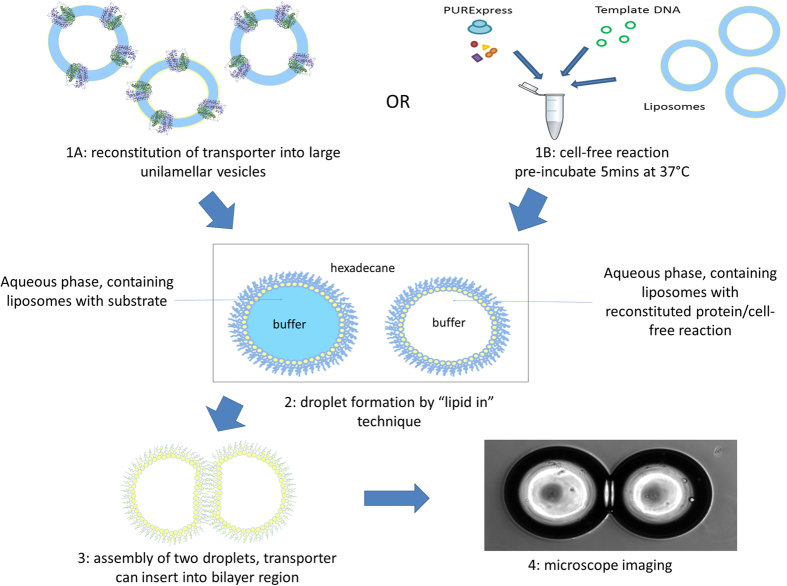
Assembling a Droplet Interface Bilayer transport system. 1. Mix aqueous phase together, comprising either A: MFS protein reconstituted into LUVs or B: cell-free reaction with added empty liposomes and template DNA. Prepare second droplet containing substrate and empty liposomes. 2. Submerge sub-microlitre droplet of the aqueous phase in hexadecane solvent bath and incubate to allow monolayer formation around each individual droplet. 3. Manipulate two droplets together so that a bilayer interface is formed between them. 4. Use microscopy to take images of the DIB and to track the transport of fluorescent substrates.

In this study we present the first example of a secondary transporter incorporated into a Droplet Interface Bilayer system resulting in active transport of a molecular substrate against a concentration gradient. The transport protein was incorporated either from pre-reconstituted proteoliposomes or expressed in the droplet using an *in vitro* cell-free expression system (Fig. 6). It has previously been reported that bacteriorhodopsin incorporated into a DIB can be used to produce uphill proton currents^17^. Here we show bulk transport and accumulation of a biomolecule. Secondary transporters, including the Major Facilitator Superfamily, are a ubiquitous class of transporters, which import and export a wide range of metabolites, toxins and other compounds into and out of cells with a high degree of substrate specificity. LacY is an extensively studied member of this family of transporters, whose activity has been previously shown to be dependent on the membrane environment both *in vivo* and in liposomes *in vitro*. The fold and topology of the protein have been shown to be altered in cells with PE-depleted membranes and in liposomes made with high concentrations of PG and cardiolipin^24^,^38^. This misfolded structure can only facilitate downhill diffusion of substrate and can no longer catalyse uphill substrate concentration. Recombinant LacY, reconstituted here into a DIB retained its activity, with transport stimulated by a proton gradient and the presence of non-lamellar lipids, and importantly including the ability to transport substrate up a concentration gradient.

The choice of lipid in an artificial membrane system can be vital. To date, most work on DIBs have used the lipid 1,2-diphytanoyl-sn-glycero-3-phosphocholine, a saturated and branched phospholipid that has been favoured especially in the field of electrophysiology for its properties of forming fluid, stable bilayers across a wide temperature range. However, many membrane proteins have specific lipid requirements for folding or function, for example the presence of charged or non-bilayer lipids. Thus, these lipids need to be successfully introduced, alongside transporter proteins, to expand the functionality of DIB networks. Here we used mixtures of synthetic lipids to strike a balance between forming stable DIBs and good protein function, by including non-lamellar DOPE as well as the charged DOPG, which also provides an environment more similar to the bacterial membrane.

The most favoured proteins used in DIB systems thus far have been various types of channel, commonly α-haemolysin, which allow for the passive diffusion of ions or small molecules across the bilayer. Transporters offer significant advantages over channels when using DIB systems for compartmentalised chemistry or synthetic biological applications. The ability to transport specific molecules in bulk across the bilayer and importantly to drive transport up a concentration gradient is an essential property of native membranes, and re-creating this *in vitro* will be a requirement for producing artificial cells. The cell-free synthesis of LacY in a droplet demonstrates that these proteins can be made and successfully incorporated into a DIB. This on board synthesis gives another potential avenue of control over the trafficking of molecules around DIB networks, which is likely to be translatable to other MFS transporters, providing a method for the selective feeding in of substrate or removal of product in compartmentalised chemical reactions. Recently, a light-activated promotor was developed that provides a mechanism for switching on cell-free production of water soluble proteins and the toxin α-haemolysin post-assembly of the DIB network^33^. Combining these sorts of development with the broader functionalisation of DIB membranes by a variety of transporters and lipids, together with the use of newer techniques for DIB network formation such as microfluidics and 3D printing provide promising paths for controlling the flow of metabolites in the next generation of artificial cell systems^39^,^40^,^41^.

## Materials and Methods

Detergents dodecyl maltopyranoside (DDM) and octyl glucopyranoside (OG) were purchased from Anatrace. Lipids 1,2-dioleoyl-*sn*-glycero-3-phosphocholine (DOPC), 1,2-dioleoyl-*sn*-glycerol-3-phosphoethanolamine (DOPE) and 1,2-dioleoyl-*sn*-glycero-3-phospho-(1’-*rac*-glycerol) (DOPG) were purchased from Avanti Polar Lipids. All other reagents were purchased from Sigma-Aldrich unless specified.

### Protein purification and reconstitution

LacY was expressed and purified as detailed in ref. 42. Briefly, LacY with a C-terminal 10-His tag was overexpressed from the pET28a vector in BL21-AI *E. coli* (Life Technologies). Cultures were grown at 37 °C in LB media until an OD_600_ of 0.8 AU then induced with 1 mM IPTG and 0.1% (w/v) arabinose until growth became stationary. Cells were harvested, cracked using a microfluidiser (Constant Systems) and the membrane fraction isolated and solubilised in buffer containing 50 mM sodium phosphate pH7.4, 200 mM NaCl, 20 mM imidazole, 10 mM β-mercaptoethanol, 10% (v/v) glycerol and 2% (w/v) DDM. The solubilised protein was loaded onto a 1 ml HisTrap HP nickel affinity column (GE Healthcare), washed with buffer containing 75 mM imidazole and 0.05% (w/v) DDM, then eluted with 500 mM imidazole. Protein-containing fractions were exchanged into 50 mM sodium phosphate pH7.4, 1 mM β-mercaptoethanol, 10% (v/v) glycerol and 0.05% (w/v) DDM using a 5 ml HiTrap desalting column (GE Healthcare).

LacY was reconstituted into large unilamellar vesicles (LUVs) as described in ref. 5. Lipids were dissolved in cyclohexane, combined in the required molar ratios then freeze-dried overnight under vacuum. The resulting lipid films were rehydrated at a concentration of 10 mg/ml (w/v) in 50 mM sodium phosphate buffer pH7.5, then passed a minimum of 11 times through a 100 nm filter using a Mini-Extruder (Avanti Polar Lipids) to create LUVs. The liposomes were pre-saturated with detergent by adding OG to a final concentration of 1.2% (w/v), before adding purified LacY at a 3000:1 lipid:protein molar ratio. After incubating at RT for 1 hour, excess detergent was removed by incubation with Biobeads (Biorad). Before use in forming droplets the liposomes and proteo-liposomes were diluted ten-fold in 50 mM sodium phosphate buffer at the required pH. For transport assays 50 μM 4-methylumbelliferyl-β-galactopyranoside was included in the droplet buffer. For monitoring pH gradients, 50 μM pyranine was included.

### Droplet Interface Bilayer formation

Droplet Interface Bilayers were prepared by the “lipid-in” method, largely as described in refs 8,9. 400 nL droplets, containing either empty liposomes or proteoliposomes were pipetted into a glass dish containing hexadecane and equilibrated for 15 mins to allow for the formation of the lipid monolayer. The two droplets were then pushed together to form a bilayer between the droplets and imaged on an IX83 inverted microscope (Olympus) with a 4x objective lens. Fluorescent substrates were detected with a DAPI excitation and emission filter set, and quantified by integrating the fluorescent signal in each droplet using ImageJ software^43^. All experiments involving droplets and DIBs were performed at 25 °C (apart from initial 5 min incubation for PURE synthesis as below).

### Cell-free expression in droplets

The PURExpress *In Vitro* Protein Synthesis Kit (New England Biolabs) contains all the proteins, ribosomes, amino acids and NTPs required for cell-free protein synthesis. A 12.5 μl reaction was set up within a droplet, following the manufacturer’s instructions, supplemented with liposomes at a final concentration of 5 mg/ml (w/v), pH7.6. 100 ng of pET28-LacY-H10 plasmid was added and the reaction incubated at 37 °C to initiate transcription. Droplets were then prepared by submerging 600 μl of either the cell-free reaction or 5 mg/ml (w/v) liposomes in 50 mM HEPES pH6.6 in room temperature hexadecane and incubating for a further 5 mins to allow for monolayer formation. Bilayer interfaces were then formed between two droplets and imaged as described above.

## Additional Information

**How to cite this article**: Findlay, H. E. *et al. In vitro* synthesis of a Major Facilitator Transporter for specific active transport across Droplet Interface Bilayers. *Sci. Rep.* **6**, 39349; doi: 10.1038/srep39349 (2016).

**Publisher's note:** Springer Nature remains neutral with regard to jurisdictional claims in published maps and institutional affiliations.

## Supplementary Material

Supplementary Information
