# Supplementary material for: In vitro synthesis of a Major Facilitator Transporter for specific active transport across Droplet Interface Bilayers
Source: Sci Rep. 2016 Dec 20;6:39349. doi: 10.1038/srep39349 (PMC5172200; doi:10.1038/srep39349)
Supplement: Supplementary Information [file srep39349-s1.pdf]

Supplementary Information

In vitro synthesis of a Major Facilitator Transporter for specific active transport across Droplet Interface Bilayers

Heather E Findlay, Nicola J Harris and Paula J Booth*

Department of Chemistry

Kings College London

Britannia House, 7 Trinity Street

London, SE1 1DB, UK

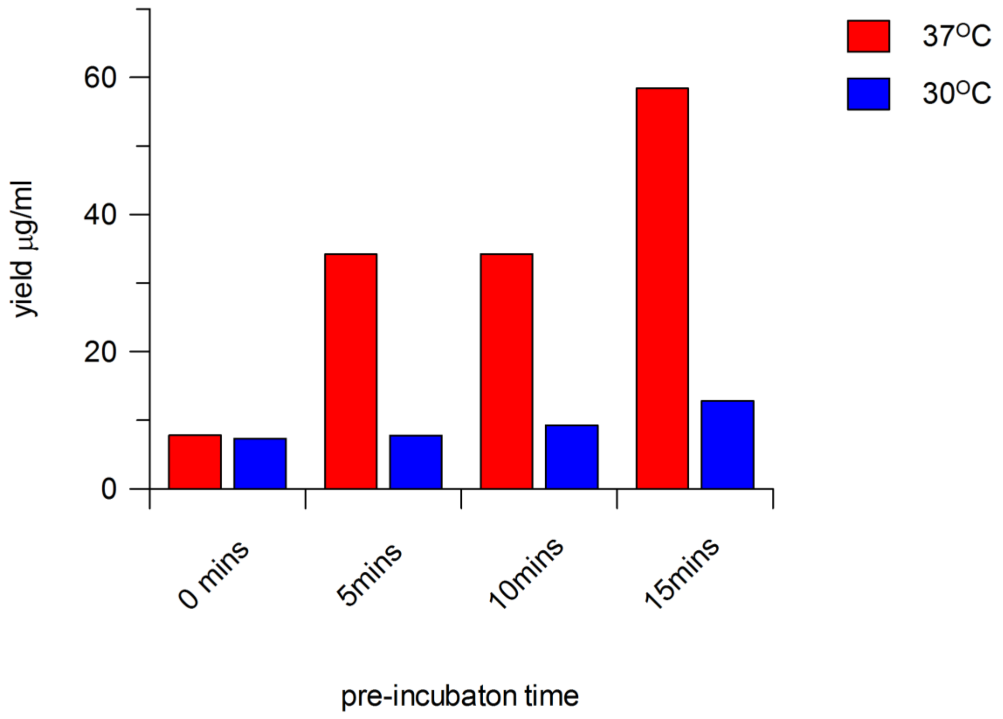

Supplementary Figure 1: Cell-free expression of LacY in PURExpress. Cell-free reactions were assembled as described for *in vitro* synthesis in DIBs, but including ^{35}S-Met and incubated for a total of 2 hours. Reactions were given a pre-incubation step of 0, 5, 10 or 15mins at either 37°C or 30°C before transferring to 25°C for the remaining time. The total yield of protein was measured by the incorporation of the radioactive methionine, quantified using a scintillation counter. A 30°C incubation made little difference to the amount of protein produced, but even a short 5 min incubation at 37°C resulted in a 7 fold increase in yield.
